# Membrane fusion, potential threats, and natural antiviral drugs of pseudorabies virus

**DOI:** 10.1186/s13567-023-01171-z

**Published:** 2023-05-02

**Authors:** Ni Ye, Wei Feng, Tiantian Fu, Deyuan Tang, Zhiyong Zeng, Bin Wang

**Affiliations:** College of Animal Science, Guizhou University, Guiyang, 550025 China

**Keywords:** Pseudorabies virus, membrane fusion process, human infection, antiviral, traditional Chinese medicine

## Abstract

Pseudorabies virus (PrV) can infect several animals and causes severe economic losses in the swine industry. Recently, human encephalitis or endophthalmitis caused by PrV infection has been frequently reported in China. Thus, PrV can infect animals and is becoming a potential threat to human health. Although vaccines and drugs are the main strategies to prevent and treat PrV outbreaks, there is no specific drug, and the emergence of new PrV variants has reduced the effectiveness of classical vaccines. Therefore, it is challenging to eradicate PrV. In the present review, the membrane fusion process of PrV entering target cells, which is conducive to revealing new therapeutic and vaccine strategies for PrV, is presented and discussed. The current and potential PrV pathways of infection in humans are analyzed, and it is hypothesized that PrV may become a zoonotic agent. The efficacy of chemically synthesized drugs for treating PrV infections in animals and humans is unsatisfactory. In contrast, multiple extracts of traditional Chinese medicine (TCM) have shown anti-PRV activity, exerting its effects in different phases of the PrV life-cycle and suggesting that TCM compounds may have great potential against PrV. Overall, this review provides insights into developing effective anti-PrV drugs and emphasizes that human PrV infection should receive more attention.

## Introduction

Pseudorabies virus (PrV) is a linear double-stranded DNA virus belonging to the *Varicellovirus* genus in the Alphaherpesvirinae subfamily of Herpesvirales. Other representative members of Alphaherpesvirinae are the varicella-zoster virus (VZV), *Varicellovirus* Bovine alphaherpesvirus 1 (BoHV-1), *Herpes simplex* virus 1 and 2 (HSV-1 and HSV-2, respectively), Marek’s disease virus (MDV), and infectious laryngotracheitis virus (ILTV). However, HSV-1 and HSV-2 belong to the *Simplexvirus* genus and MDV and ILTV to *Mardivirus* and *Iltovirus*, respectively [1]. The PrV genome is approximately 140 kb, with a GC content averaging 74%, and is predicted to encode more than 70 different proteins. The viral genome structure includes long- and short unique regions and internal- and terminal repeats [2, 3]. Genes on the PrV genome encode structural and virulence proteins, enzymes, and proteins related to viral invasion, replication, and release [4].

As the causative agent of Aujeszky’s disease (AD) or pseudorabies (PR), PrV has a broad host range, including domestic animals [5], wild animals [6, 7], and experimental animals such as rabbits and mice [8]. Although PrV infection is rare in humans, it is a potential threat to public health. PrV causes acute encephalitis, endophthalmitis, and retinal vasculitis in humans [9–11]. Pigs are the natural PrV hosts, and this viral infection is primarily reflected in respiratory symptoms in older pigs, fetus death, abortion in pregnant sows, or both. In young piglets and more susceptible species, PrV infection is often fatal as it leads to central nervous system disorders [12, 13]. PrV can also establish a lifelong latent infection in the peripheral nervous system. In pigs stimulated by external stimuli or with weakened immunity, latent PrV was reactivated from peripheral neurons, replicated, and produced new progeny viruses that led to the re-emergence of pseudorabies [14]. This ability of PrV brings challenges to eradicating pseudorabies.

This disease causes considerable economic losses in the swine industry every year [15], and vaccination using classical attenuated live PrV or inactivated PrV vaccines has been one of the most important methods to prevent and control pseudorabies [13]. Many European countries, New Zealand, and the United States rely on strict national eradication programs based on mandatory vaccination, culling positive pigs, and establishing PrV-free pig herds. AD has been eradicated from domestic pig populations [16], but PrV is still found in wild boars and other wild animals [17, 18]. Thus, PrV is still a risk for outdoor pig farms in these countries. In China, measures are being taken to eradicate AD. The traditional Bartha-K61 vaccine is the most widely used in China. Before 2011, this vaccine was able to control pseudorabies outbreaks, but since late 2011, Bartha-K61 has been unable to provide complete protection against PrV strains [19]. The emergence of mutant strains is undoubtedly a considerable obstacle to preventing PrV. Thus, developing PrV vaccines based on the epidemic strains is crucial, which can prevent or eradicate pseudorabies. Furthermore, novel anti-PrV drugs are required to prevent PrV-induced latent infection and decrease the virus pathogenicity. In addition to vaccines and drugs, one potential strategy to prevent pseudorabies is to develop antibodies or blockers targeting the biomolecules involved in PrV infection, such as receptors and ligands.

## PrV invasion

The mature PrV virion comprises four structural elements: the linear DNA genome, a protective icosahedral capsid, the tegument, and the envelope. The envelope is derived from the membrane structure of the host cell infused with virus-encoded proteins and participates in virus entry, egress, cell-to-cell spread, and immune response. These proteins include gB, gC, gD, and gE and more than 11 proteins are modified by N- or O-linked sugars and non-glycosylated proteins UL20, UL43, and US9 [4, 20]. The tegument, located between the capsid and the envelope, contains outer and inner tegument proteins [21, 22]. The outer tegument protein preferentially associates with viral membranes, whereas the inner tegument protein adjacent to the capsid forms a capsid-tegument complex after virus entry into the cell. This capsid-tegument is closely related to PrV transport along microtubules into the nucleus. Compared with other enveloped viruses, PrV binding and access into target cells require the concerted actions of at least five glycoproteins (gC, gD, gL, gH, and gB). A detailed understanding of PrV-cellular membrane fusion at the molecular level is essential for developing new therapeutic strategies, especially receptor and ligand-targeting drugs to block virus invasion and transmission. The invasion mechanism of HSV alphaherpesviruses has been extensively described and often used as baseline research on other alphaherpesviruses. However, in addition to the conserved characteristics, alphaherpesviruses have unique features. Thus, in the following section, we explore the invasion mode of PrV.

### PrV adhesion and gD

Virus entry into cells results from the coordinated interaction between viral proteins and cellular receptors. The initial PrV attachment to the cell is weak and does not trigger the virus entry. At this stage, PrV gC binds to the heparan sulfate (HS) receptor on the cell membrane [23], and then gD binds firmly to the cell receptor nectins (mainlly nectin-1) [24]. For most alphaherpesviruses, gD is an essential, non-conserved receptor binding protein that affects the tropism of the alphaherpesviruses to cells, although VZV does not encode gD [25]. The PrV gD structure includes an immunoglobulin variable (IgV)-like core wrapped by N- and C-terminal extensions [26]. The gD homologs of HSV, BoHV-1, and PrV share only 22–33% amino acid identity, indicating that they have different entry receptor and binding affinities. Entry receptors, nectin-1/2, herpesvirus entry mediator (HVEM), HS 3-O-sulfonated derivatives for HSV, nectin-1/2 for PrV, and nectin-1 for BoHV-1, have been identified [27]. Human nectin-1 shares 96% amino acid identity with porcine nectin-1. Although PrV gD exhibits similar affinity for both human and porcine nectin-1 [26, 28, 29], the PrV gD binding affinity for human nectin-1 is ten-fold higher than that of HSV-1 gD [26, 27], which may explain human PrV infection. Despite the low amino acid homology of PrV and HSV gDs, most of their secondary structure elements are similar. However, some features such as the PrV gD C-terminal extension contains an extra α2’ helix, the steric position of the α1’ helix in PrV, and HSV gD structures are different. Furthermore, compared with the HSV gD N-terminal loop (N-loop), the shorter PrV gD N-loop cannot form the hairpin structure necessary for HVEM binding. This inability to form the hairpin structure is consistent with the fact that PrV cannot utilize HVEM as a cellular receptor [26].

The PrV gD/swine nectin-1 complex structure has been recently identified [26]. First, PrV gD recognizes and engages the swine nectin-1 CC’C’’FG sheet through str2 and its flanking loops in the N-terminal extension, the α1’ helix in the IgV-core, and helices α2’, α3’, α3, and the α3’/α3 loop in its C-terminal extension. The N-loop is then reoriented upon binding as two aromatic residues in this structure, F11 and W22, are projected outwards, forming a hydrophobic interaction with nectin-1 amino acids K61, Q64, I80, N82, M85-S88, and F129. F11 undergoes further conformational changes after receptor binding. The major forces for stabilizing the N-loop orientation in the complex structure may result from the interactions of F11 with nectin-1 K61 and F129. Before binding to the receptor, gD monomers form dimers through disulfide bonds, likely stabilizing the C-terminus conformation [30]. The membrane-proximal loop of the gD ectodomain (proposed as the pro-fusion domain, PFD) and the conserved Pro291/Trp294 in the gD C-terminus are vital in gD-mediated alphaherpesvirus entry [30–32]. The PFD length affects the PrV gD affinity for nectin-1, with a short PFD leading to higher affinity [26]. The HSV PFD pre-locates in a position that blocks the binding of gD with its receptor. However, the controlled PFD displacement exposes the locked receptor-binding site allowing gD to recognize and bind to its receptor [30]. Nectin-1 homologues and heterodimer formation are the basis for nectin-1 adhesion [33]. Because the affinity of PrV gD and human nectin-1 is higher than the nectin-1 self-affinity [26, 34], PrV gD might destroy nectin-1 dimer formation by competing with nectin-1 and changing receptor presentation on target cells. Furthermore, gD may have evolved from an essential to a non-essential infection protein. While HSV-1 requires gD for cell entry and direct cell-to-cell spread [35], PrV gD is associated with cell penetration but is dispensable in direct cell-to-cell spread [36]. PrV gD is reportedly not a core fusion protein; gD-negative PrV mutants (PrV-gD^−^ Pass) remained infective with compensatory mutations in gB and gH, both in vivo and in vitro [36, 37]. Nevertheless, the host range of PrV-gD^−^ Pass changed as this mutant virus was unable to infect pigs but was fatal for mice [37].

### The gH/L complex–a virus and cellular membrane fusion regulator

The heterodimeric complex formed by the integral membrane gH plus the anchorless gL and the homotrimeric gB jointly participate in the core PrV envelope fusion mechanism with the cellular cytoplasmic membrane. Moreover, without the gH/L complex, gB cannot function as a fusion protein [38]. Although gH/L acts as a regulator to transmit the fusion signal to gB [39], the fusion speed and rate are governed by gH/L, especially gH, but not gB [40]. As a type I transmembrane protein, gH has a small cytosolic tail and a large N-terminal ectodomain. Within the same virus subfamily, gH shows substantial variability in length and sequence, especially in its N-terminal. Additionally, compared with other herpesviruses, the PrV gH ectodomain is shorter and more compact [41]. According to the gH/L crystal structures of the Epstein-Barr virus (EBV, gammaherpesvirus) [42], HSV-2 [43], VZV [44], and PrV gH core fragment structure (residues 107 to 639) [41], gH/L (or gH alone) has no typical fusion protein features. Instead, they present a common folding structure and low sequence conservation, serving as a regulator in gB fusion activity [45]. The ectodomain comprises a predicted signal peptide (SP) and ectodomain parts I, II, III, and IV. The PrV gH domain I is formed by the N-domain in specific association with gL [46] and is similar in size to EBV domain I but half the size of HSV domain I [41].

In recent HSV studies, the physical gD to gH/L binding occurred within the gH/L ectodomain. Furthermore, the gD interaction sites on gH/L were the gH N-terminus and the gL C-terminus, more specifically, close to gL residue 77 [47]. gD also carried composite-independent binding sites for gH/L and gB, both partly located in the C-terminal pro-fusion domain [48]. The putative gH/L binding sites on HSV gD have been identified as independent to those used for HSV receptor binding, but no interaction was found between gB and gD or gH/L [39]. Monoclonal antibodies targeting gD-gH/L binding sites are also being developed to block the virus fusion [49]. However, more studies on PrV are needed to reveal whether its gD-gH/L binding site is similar to that of HSV. Domain II contains a β-sheet (named “fence” as it separates the domain from the rest of the molecule) and a syntaxin-like bundle (SLB) of three α-helices, which resembles functionally relevant elements in eukaryotic fusion proteins in the syntaxin family. The SLB structure and flexibility are relevant to the maturation and PrV gH membrane fusion activity [50, 51]. Domain III consists of eight consecutive α-helices (α6–α13). Conserved proline^438^ assists in bending at the end of α-helix 11, and when proline^438^ switches to serine, the formation of the cysteine^404^-cysteine^439^ disulfide bond is impaired, affecting cell-to-cell spread and fusion activity [52]. The flap and N-linked carbohydrate moiety, which covers an underlying patch of hydrophobic amino acids in the membrane-proximal domain IV, have attracted much research. A modest conformational change in moving the flap might be required to expose the hydrophobic patch for membrane interaction during the fusion [53]. Interestingly, the IV domains of PrV and HSV-1 gH exhibit functional conservation [46]. A chimeric gH, comprising PrV I, II, and III domains and HSV-1 IV domain showed considerable fusion activity in the presence of PrV gB, gD, and gL; Along with PrV gB, gD, and HSV-1 gL, but not PrV gL, another chimeric gH (consisting of PrV II, III, and IV domains and HSV-1 I domain) retained limited functionality, and the PrV II and III domains might be functional units that cannot be replaced [46].

The gH transmembrane domain (TMD) and cytoplasmic domain (CTD) functions differ in PrV and HSV’s fusion processes. In PrV, gH CTD can decrease membrane fusion activity but is dispensable for gH function during virus infection. In contrast, gH TMD is essential for triggering fusion and highly relevant for the PrV gH function. PrV gH lacking the TMD, membrane-anchored via a lipid linker or comprising the PrV gD TMD were unfunctional [54]. However, a transient assay has shown that soluble forms of HSV gH/L were sufficient to induce gB-mediated cell-to-cell fusion [55].

The anchorless gL plays a chaperonin protein-like function and is noncovalently linked to the gH ectodomain. gH maturation and processing require the presence of gL in herpesviruses such as HSV-1 or VZV [56–58]. However, in PrV, gL is dispensable for correct folding, transport, or gH virion localization but necessary for infectivity. In the absence of gL (PrV-△gLβ mutant), PrV gH can be detected at the cell surface, but the mutant is capable of limited cell-to-cell spread. Therefore, gL cannot solely play a chaperonin-like function in PrV [59, 60]. Furthermore, using the limited spread of the PrV-△gLβ mutant, a gDH fusion polypeptide (the gD N-terminal 271 residue was fused to the gH C-terminal 590 residue) containing a receptor binding domain of PrV gD has been isolated in serial cell passaging, which can compensate for the essential gL function in the entry and PrV cell-to-cell spread [61]. Additionally, in PrV-△gLPassB4.1, a mutant lacking gL replicated effectively because a combination of point mutations in gH (namely gH^B4.1^, mutation points: L^70^P and W^103^R in the N-terminus) and gB (namely gB^B4.1^, mutation points: G^672^R and deletion of lysine at position 883) compensated for the gL function [62]. A defective mutant incapable of cell entry and spread, PrV-gH^32/98^K (deleted codons 32 to 97 in the gH N-terminus) was unable to bind gL, resulting in impaired gL processing and rapid secretion of mature gL. The replication and entry competence of pPrV-gH^32/98^K can be restored by gB^B4.1^ rather than the wild-type gB, but entry is considerably delayed [60]. In summary, PrV gL and the gH gL-interacting domain are not strictly required for membrane fusion because gH and gB mutations can compensate for their absence, suggesting that gB and gH are the core proteins in PrV fusion.

### gB-the fusion protein

The critical core fusion protein of PrV, gB, is the most conserved envelope glycoprotein of herpesviruses and belongs to class III fusion proteins. Unlike most class III fusion proteins, which display a short and unstructured cytoplasmic tail activated by low pH and are critical for fusion, the alphaherpesviruses’ gBs have an extensive and structured CTD (90 amino acids in PrV gB) [63, 64]. Thus, PrV gB is not an autonomous fusogen but requires activation by its partner gH/L to drive pH-independent membrane fusion [65]. Signals from gH/L trigger a metastable prefusion state gB on the viral membrane, and gB undergoes a conformational change exposing the initially buried hydrophobic regions for interactions with the target membrane, resulting in the fusion of cell and viral membranes. The prefusion conformation of PrV gB remains elusive, while the structure of the post-fusion conformation gB ectodomain has been shown to display trimeric, elongated, rod-like molecules, with each monomer comprising five domains (I to V). PrV gB ectodomain shares 50.1, 25.6, and 25.3% amino acid sequence identity with HSV-1, human cytomegalovirus (betaherpesvirus), and EBV gB, respectively [66]. The CTD, TMD, and adjoining gB membrane proximal region (MPR) play critical roles in regulating extracellular domain fusion activity. The carboxy-terminally truncated derivative of PrV gB (stably expressed in cell lines), PrV gB-008 (gB protein lacking the putative internalization signals and the C-terminal α-helical domain), showed enhanced fusogenic activity, whereas gB-007 (gB protein lacking both α-helical domains) was unable to incorporate into gB-negative PrV (PrV-gB(-)) for full infectivity. However, gB-007 restored the direct viral cell-to-cell spread of PrV-gB(-). Lacking the entire CTD, the protein lost functionality [67], indicating that CTD is closely related to gB functionality. In another study, after serial passages in cell culture, PrV-007Pass gB with a compensatory mutation in the ectodomain was obtained, and it was efficiently incorporated into the viral envelope, restoring the infection ability of PrV-gB(-) [68]. The gB CTD acts as an inhibitory clamp to restrict the gB ectodomain rearrangement stabilizing the gB prefusion structure. Without CTD, the gB ectodomain remains in a post-fusion state and cannot perform its function, affecting gH/L and gB signaling. Residue V831 within the HSV gH cytoplasmic tail reportedly wedges into the pocket above the interface between the gB CTD adjacent monomer to push the monomer apart, releasing the inhibitory clamp [69]. The above activation mechanism may also exist in PrV. A chimeric gH (domains I, II, and III of PrV gH plus the IV domain, TMD, and CTD of HSV-1 gH) could trigger PrV gB effectively [46]. HSV gH efficiency might differ from PrV gH for PrV gB activation due to its insertion into the pocket. The gB CTD plays an essential role in maintaining gB prefusion, and a regulatory element in gB domain V controlling the fusogenic gB conformational change has been identified. The PrV-gB^△CTD2^ mutant (gB lacking 60 C-terminal amino acids) showed defective entry but was capable of cell-to-cell transmission. A PrV-gB^△CTD2^Pass mutant restoring viral infectivity was obtained using this exceptional phenotype after serial passaging in vitro. Analysis of this variant (PrV-gB^△CTD^Pass) revealed that a single mutation in a conserved domain V residue (N735S) could compensate for the absence of CTD and support the gH/L-independent viral entry. Similar to CTD, the mutated domain can stabilize the gB ectodomain in a fusion-active conformation. Additionally, a short conserved α-helical structure in domain V (residues 728 to 742) was defined as the molecular switch for gB pre-to-post fusion. As in PrV N735, a conserved amino acid mutation in ILTV and BoHV-1 (BoHV-1 N742S and ILTV Q684S/N685S, respectively) but not in HSV-1, can also compensate for the absence of CTD and lead to gB autonomous fusogenicity, independent of the gH/L complex. This finely tuned functional tandem between fusion regulatory domains within gB may be common in alphaherpesviruses PrV, ILTV, and BoHV-1 but not in HSV-1, which may have different regulatory mechanisms [63].

The role of the regulatory TMD and MPR ectodomains has been studied in HSV and PrV. The gB TMD participates in membrane anchoring and the late fusion stage. Therefore, when HSV-1 gB TMD is replaced by a lipid anchor, the fusion can only proceed halfway [70]. The PrV gB MPR has 50 residues and acts as a shield between the gB fusion loops (FLs) and the target cell membrane before fusion occurs [66, 71]. Upon receiving the fusion signal, the gB structure undergoes rearrangement, and the appropriate movement of the MPR exposes the FLs and initiates membrane fusion. The membrane-interacting regions of the fusion protein contain abundant hydrophobic and aromatic residues for insertion into the outer leaflet of the cell membrane [72]. In PrV, the FLs (FL1 and FL2) in domain I are exposed at the base of the gB molecule. First, Phe275 at the tip of FL2 is deeply inserted into the hydrocarbon core of the phospholipid bilayer. Then, Trp187, Tyr192, and Tyr276 on the side chains of FL1 and FL2 are inserted shallowly into the membrane, forming a fusion edge that catalyzes the fusion process between PrV and the cell membrane [66]. Developing protective monoclonal antibodies (mAbs) against PrV gB, 1H1 mAb acting on the gB domain I, showed broad neutralizing activity without the complement participation, presumably by inhibiting PrV fusion to the cell membrane [73]. The other 14 mAbs identified in this study required complement participation and acted on domain IV (crown region). These results indicate multiple B-cell epitopes in the gB crown and base regions.

**Figure 1:**
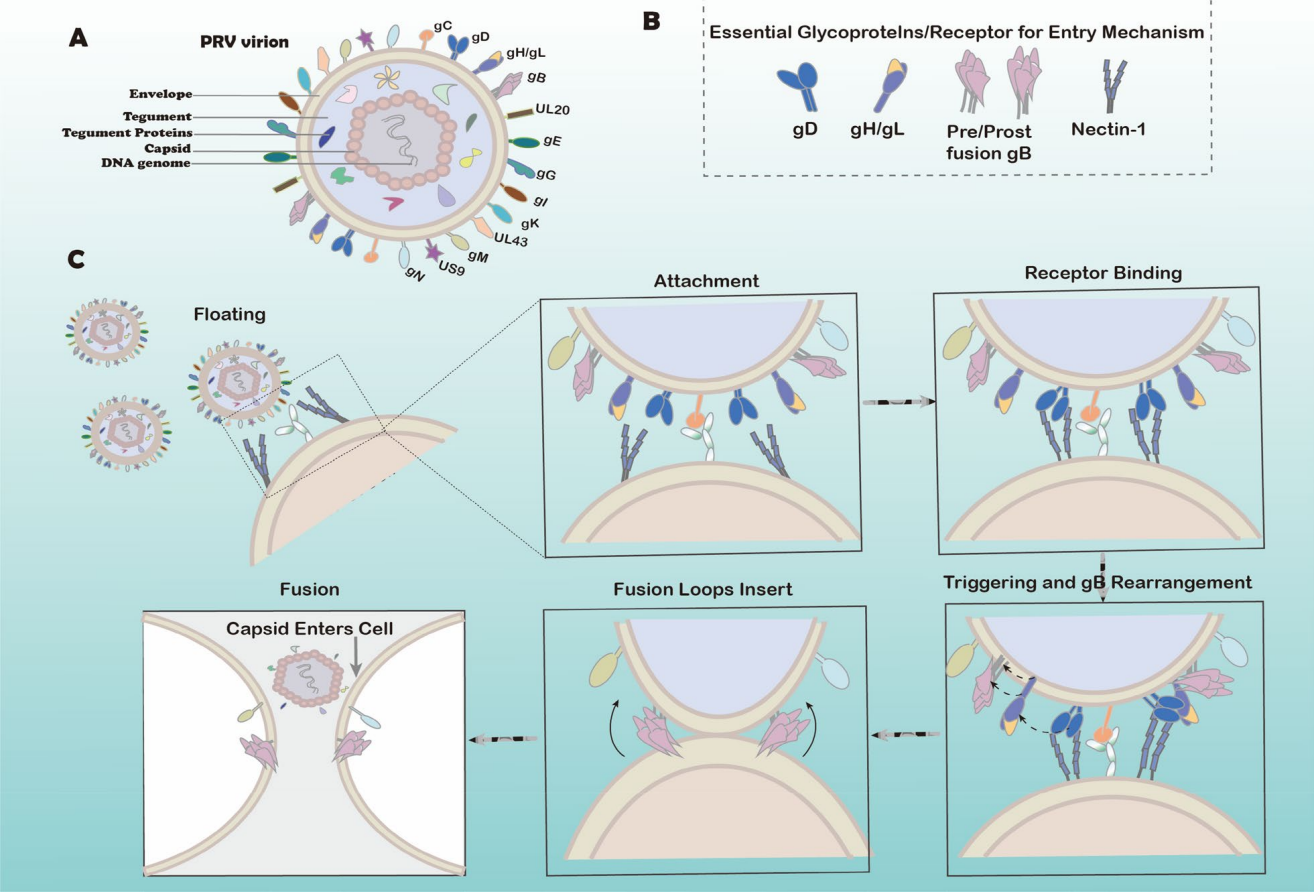
**PrV entry mechanism through membrane fusion.** **A** PrV virion simulation diagram. PrV virions are composed of four structural elements: a lipidic envelope containing non-glycosylated trans-membrane proteins UL20, UL43, US9, and glycosylated membrane proteins gB, gC, gD, gE, gG, gH, gI, gK, gL, gM, and gN; a dense protein tegument layer, generally classified as outer and inner tegument proteins; an icosahedral protein capsid; and a linear double-stranded DNA genome. **B** The essential viral glycoproteins gD, gH/L, gB, and host receptor nectin-1 for the PrV entry mechanism. **C** The viral-cell membrane fusion process. Attachment: PrV binds with HS via its gC; this step enhances entry but is not required for fusion. Receptor binding and attachment stabilization: gD binds to its nectin-1 receptor to stabilize PrV binding; the C-terminus of the gD ectodomain exposes the receptor binding site, completing gD binding to nectin-1. This transmits a signal to activate gH/L, which may come directly from the C-terminus moving along the gD ectodomain or from other interaction sites exposed by the C-terminus moving and physical interaction between gD and gH/L. Triggering and gB rearrangement: gH/L transmits this signal via the physical binding of gH/L and gB ectodomain and the gH CTD interaction with the gB CTD to trigger core fusion protein gB. Upon triggering, the prefusion gB conformation undergoes rearrangement, emerging as a more stable post-fusion gB conformation. Fusion loop insertion: the fusion loops of post-fusion gB insert into the target cell membrane. Fusion: as gB shrinks, the fusion loops gradually drive the cell membrane closer to the gB TMD completing the viral-cell membrane fusion. Finally, the capsid associated with inner tegument proteins enter the target cell.

In the current model of alphaherpesviruses entry, PrV invades cells through three basic steps: initial adsorption, binding of the entry receptor, and membrane fusion (Figure 1). However, in the well-studied HSV, multiple receptors that can interact with gH/L or gB have also been found, such as the gH alphavbeta3-, alphavbeta6-, and alphavbeta8-integrins receptors [74, 75], the paired immunoglobulin-like type 2 receptor-α, myelin-associated glycoprotein, and non-muscle myosin heavy chains IIA and IIB of gB [76–79]. HSV-1 and BoHV-1 entry is reportedly inhibited by the soluble form of their gBs, which implies that the receptors for gB may be directly involved in cell invasion [80, 81]. Compared with HSV, there is less information on PrV gH/L and gB receptors. In addition to the membrane fusion process mediated by gB, another fusion pathway with receptor-mediated or auxiliary involvement may be involved in PrV fusion, but further studies must be done.

## Current status of PrV infection in humans

Although pigs are the natural PrV hosts, the virus can infect other animals, including cattle, sheep, mink, mice, rabbits, cats, and dogs. PrV can reportedly infect humans, and contact hosts history of the human suspected case included cats, dogs, and cattle infected with PrV; In contrast, a previous study confirmed that humans are not susceptible to PrV or at least to the strains used in the tests [82]. At the end of 2011, a PrV outbreak occurred in pig farms immunized with the Bartha-K61 vaccine in many regions of China. So far, several PrV variants with enhanced virulence have been isolated throughout the country. Since 2017, more than 28 human cases of PrV infection have been reported in China [83–85], and the PrV strain hSD-1/2019 was first isolated from human cerebrospinal fluid (CSF) [9]. Whether PrV can infect humans has been controversial because previous reports were not based on virus isolation. The diagnoses of the above cases were mainly based on the PrV nucleotide detection with metagenomic next-generation sequencing (mNGS). PCR and serological tests further validated some. We selected 16 of these 28 cases, confirmed by mNGS and had relatively complete and detailed case presentations (Table 1).

**Table 1:** **Case reports of PrV infection in humans**

Case	Age/Sex/Occupation	Contact history	Clinical manifestation	Antiviral treatment*	Clinical outcomes	References
1	43/male/swineherd	Pigs	Fever, loss of consciousness, limb convulsions, mood abnormalities, eye deviation, urinary incontinence	Acyclovir, foscarnet	Psychiatric disorders, intermittent blurred vision, and impaired cognitive function	[84]
2	51/female/housewife	Pork	Headache and drowsiness, fever, delirium, seizures, abnormal behavior	Acyclovir	Remained unconscious with the metal tracheostomy tube, a modified Rankin Scale of 5, and a Glasgow coma scale score of 7	[93]
3	68/male/swineherd	/	Fever, recurrent seizures, epileptics and respiratory failure	Acyclovir	Discharged unconscious and ventilator-dependent; the patient died 1 week after discharge	
4	54/male/slaughterhouse worker	Several minor cuts during the dissection of the dead pig	Cough, fever, fuzzy consciousness, fecal, headache, and urinary incontinence	Acyclovir, ribavirin	The prognosis was extremely poor as the patient was unable to wean off mechanical ventilation	[94]
5	25/male/veterinarian	PRV-infected pigs	Fever, headache, memory loss, seizures, impaired consciousness	Ganciclovir, foscarnet	Permanent eye damage and blindness	
6	35/male/pig butcher	Pigs and pork	Fever, respiratory failure, headache, memory loss, seizures, impaired consciousness	Acyclovir, foscarnet	Mild memory impairment	[9]
7	49/male/pig butcher	Pork	Fever, respiratory failure, Headache, memory loss, seizures, impaired consciousness	Ganciclovir, foscarnet	Minimally consciousness	
8	43/male/veterinarian	Diseased pigs	Fever, respiratory failure, headache, memory loss, seizures, impaired consciousness	Acyclovir, foscarnet	Persistent vegetative status	
9	44/male/pig pork vendor	Direct contact with pork with injured fingers	Fever, seizures, consciousness disorder, encephalitis	Ganciclovir, acyclovir	Could perform eye movements and simple body movements in response to verbal commands, discharged after 6 months in hospital	[95]
10	50 /male/pig slaughterer	Finger was kicked by a pig	Fever, headache, visual disturbance, convulsions, urinary and stool incontinence	/	Slow responses with occasional seizures	
11	50 /female/pork cutter	Hand injury	Fever, memory loss, coma, convulsion, respiratory failure	/	Difficulty breathing; required ventilator support	
12	43 /male/sick pig handler	Hand injury	Fever, extremity tremors, respiratory failure	/	Followed simple instructions but showed blurry vision	
13	59/male/pork cutter	Hand injury at work	Fever, extremity convulsions, respiratory failure	/	Difficulty breathing and required ventilator support	[86]
14	50/male/pork cutter	No injury	Fever, extremity convulsions, altered mental status, respiratory failure	/	Slow responses and vision loss	
15	43/male/veterinarian	Hands punctured during the autopsy process of dead swine	Fever, headache, tonic–clonic seizures, coma	Ribavirin, acyclovir	Dependent on tracheostomy and gastrostomy tubes, needed further neurological function rehabilitation	[96]
16	46/female/swineherder	Sewage from a pig farm spilled into the eyes	Fever, headache, visual impairment	Acyclovir, valacyclovir	Lost the vitreous of the right eye	[11]

*This list reflects only antiviral drugs, not including other drugs such as antibiotics, anti-epileptic drugs, and drugs regulating immunity provided to patients; / Data not provided in the reference.

The patients included swine herders, veterinarians, pig butchers, and people with occupations mostly related to pig production and sales. The clinical symptoms were not restricted to fever, headache, and cough in the prodromal stage, and seizures, coma, respiratory failure, and other symptoms in the dominant period [83]. Antiviral therapy, including acyclovir, ganciclovir, and foscarnet, is essential for patients, and intravenous human immunoglobulins, glucocorticoids, anti-epileptics, and antibiotics were administered according to clinical symptoms [9, 86]. The outcome of PrV-caused encephalitis or endophthalmitis is poor, and there is no specific drug to prevent the progress of human encephalitis or endophthalmitis caused by PrV. However, most patients have residual symptoms, and the number of deaths is small [9, 87]. The contact history of patients reveals that human infection with PrV is related to occupational exposure. In addition, PrV transmission from animals to humans may occur via companion animals, and suspected cases of PrV transmission from dogs or cats to humans have been reported [82]. In addition to the growing number of people keeping companion animals, cats and dogs are at high risk of PrV infection by eating PrV-infected raw meat or preying on wild animals [88, 89].

A study detected anti-gB and anti-gE positive samples in dog serum collected at pet hospitals and anti-gB positive samples in the dog serum from pig farms. Commercial vaccines for PrV may be a hidden risk for dogs and owners [90]. 60-day-old beagle dogs infected with the commercial vaccines Bartha-K61 or HB98 strains, which are widely used in the Chinese swine industry, could shed the virus via buccal/nasopharyngeal/anal swabs, and the Bartha-K61 strain could be transmitted among dogs. Moreover, PrV has been isolated in hunting dogs in many European countries where PrV has been eradicated from domestic pigs. Genetic analysis of PrV strains showed that in addition to genotype I, frequently reported in Western Europe, there is also genotype II of Asian origin, suggesting that wild animals are potential sources of infection [91]. The sequence alignment analysis of hSD-1/2019 indicated a close phylogenetic relationship with HN1201 and HLJ8 strains and the BJ/YT strain isolated from a Yorkshire terrier dog [9]. Combining the above studies and considering the closeness between humans and dogs, more studies are needed to confirm whether there is a relation between human PrV infection and exposure to dogs.

A retrospective PrV seroepidemiological investigation was carried out on 1335 serum samples from patients with encephalitis from nine provinces in China. The enzyme-linked immunosorbent assay results showed that the PrV-positive rates were 12.16, 14.25, and 6.52% in 2012, 2013, and 2017, respectively [92]. This result suggests that PrV may be the pathogenic agent in some undefined human encephalitis. PrV and HSV belong to the Alphaherpesvirinae subfamily and share some biological characteristics and infection mechanisms. Humans are the natural host of HSV, and silent infections are extremely common in the population. Moreover, the binding affinity of PrV gD to the human nectin-1 receptor is ten times higher than that of HSV gD [26, 27]. These characteristics suggest that PrV is highly likely as a potential zoonotic pathogen.

## Natural plant extracts as potential anti-PrV drugs

Despite the global vaccination programs, many countries still report PrV infections in domestic pigs and wild animals, and latent infections may be present. Moreover, there is no approved effective drug to treat diseases caused by PrV in swine, humans, and other animals. Acyclovir is one of the most effective treatment options against HSV-1 and VZV. However, after acyclovir treatment, human viral encephalitis caused by PrV showed limited or slight improvement [9]. Currently, PR prevention and control are mainly based on vaccines in the pig industry. Since 2011, the classic Bartha-K61 vaccine is not entirely effective against some PrV variants, which hinders the eradication of PR in China. Gene-edited vaccines based on PrV epidemic strains have been widely studied [97] and include single- [98], double- [99], triple- [100], four- [101], and five gene-deleted [102] vaccines with good development prospects. Furthermore, some of these vaccines have been licensed [103]. Compared with many studies on PrV vaccines, research on specific drugs, mAbs, and small molecule PrV inhibitors must be supplemented. PrV risks should be taken seriously, especially regarding mutant strains. Antiviral strategies are required for swine and humans to prevent and treat PrV-induced diseases and decrease the virus’ pathogenicity. The following section mainly describes the plant extracts used against PrV in traditional Chinese medicine (TCM).

### Flavonoids

In the history of human civilization, TCM has been widely used in China for thousands of years. Natural drugs have less resistance and side effects. Their antiviral effects include direct action on the virus and target cells and enhanced immunity to protect the body from virus damage. Natural products have a wide structural diversity and are essential sources of new therapeutic drugs. After years of research, TCM has also progressed in resisting PrV (Table 2). Epidemiological evidence suggests that dietary flavonoids are beneficial to human health [104, 105].

**Table 2:** **Potential traditional Chinese medicine ingredient against pseudorabies virus**

Name	PRV strains	Model	Inhibitory phase	Mechanism of action or pathway	References
Curcumin	Rong A strain	Hippocampal neurons (rat)	/	Exhibits a neuroprotective effect against PrV infection via regulating the BDNF/TrkB pathway	[109]
EGCG	XJ5 strain Ra strain	PK15 B6 cells, BALB/c mice	Multiple steps	Blocks PrV adsorption, entry, and replication in vitro	[132]
Kaempferol	Ra strain	KM mice	Early phase	Reduces IE180 gene expression level and inhibits viral replication in vivo	[125]
Quercetin	HNX strain	Vero cells, BALB/c mice	Early phase	Occupies most of the nectin-1 binding regions on gD Reduces the ROS secretion and alleviates oxidative stress	[129] [131]
DMY	PRV	PK-15 cells	Early phase	Interferes with virus invasion and decreases pyroptosis	[133]
IBC	PRV	PK-15 cells	Late stage	Inhibits the late stage of viral replication by blocking PrV-mediated cell-to-cell fusion	[134]
FEA	PRV-GXLB-2013 strain	RAW264.7 cells	Late stage	Attenuates PrV-induced inflammation by inhibiting the NF-κB and MAPK pathways’ activation	[135]
PGPS_t_	SX strain	PK-15 cells	early phase	Inhibits the accumulation of autophagosomes caused by PrV infection and upregulates the Akt/mammalian target of the rapamycin signaling pathway	[136]
HRP	XJ5 strain	PK-15 cells	Early phase	Reduces malondialdehyde and ROS levels and increases superoxide dismutase activity	[141]
PLP	XJ5 strain	PK-15 cells	Early phase	Reduces viral adsorption and entry and intracellular ROS levels and may have electrostatic interaction with cell surface proteins	[145]
Resveratrol	Rong A strain, Bartha K-61,	PK15 cells, ICR mice, piglets	/	Inhibits IE180 transcriptional activation activity by targeting the Thr601, Ser603, and Pro606 sites of IE180 protein	[148–151]

/ Data not provided in the reference. BDNF, brain-derived neurotrophic factor; DMY, dihydromyricetin; EGCG, (-)-epigallocatechin-3-gallate; FEA, an ethyl acetate fraction of flavonoids from *Polygonum hydropiper L.*; HRP, *Hippophae rhamnoides* polysaccharide; IBC, isobavachalcone; PGPSt, *Platycodon grandiflorus* polysaccharides; PLP, *Plantago asiatica* polysaccharide; ROS, reactive oxygen species.

Brain-derived neurotrophic factor (BDNF) is highly involved in neuronal plasticity, protection, and mitochondrial transport and distribution regulation [106]. Increasing BDNF levels protect neurons from insults. This neuroprotective effect of BDNF is achieved primarily by binding to transmembrane tropomyosin-related kinase B (TrkB) on the mitochondrial membrane [107, 108]. PrV infection induced oxidative damage and decreased BDNF levels in rat hippocampal neurons, while 10 µM curcumin improved the viability of PrV-infected (a titer of 3.06 × 10^6^ TCID_50_) hippocampal neurons [109]. Curcumin, a natural phenolic compound found in *Curcuma longa L.*, has anti-inflammatory, neuroprotective, and antiviral activity [110, 111]. Curcumin can cross the blood–brain barrier and is a promising agent for treating neurodegenerative diseases [112]. The mechanisms underlying the curcumin-mediated neuronal protection against PrV infection is reflected in that curcumin can inhibit the decrease of BDNF and reduce oxidative stress, apoptosis and mitochondrial damage through the BDNF-TrKB pathway [109]. Disease caused by HSV and PrV is characterized by recurrence, and some patients with HSV or PrV infections will need lifelong treatment. Therefore, drug toxicity and residues must be considered in developing new anti-PrV drugs. Curcumin was classified as a “Generally Recognized as Safe” compound and confirmed with excellent tolerance in doses up to 12 g/day in humans [113]. Nevertheless, curcumin has weak bioavailability [114]. Efficient drug delivery systems were developed to solve this shortcoming, such as curcumin nanocrystals [115], nanoliposome-encapsulated curcumin, and curcumin proniosomes [116, 117], and oral supplements with improved curcumin absorption and bioavailability are currently accessible on the market [118, 119].

Kaempferol is a flavonoid found in medicinal plants such as *Ginkgo biloba*, *Tilia* spp., *Equisetum* spp., *Moringa oleifera* and edible plants, including tea, broccoli, cabbage, and leeks [120]. Kaempferol shows anti-inflammatory [121], antioxidant [122], antiviral [123], and other pharmacological activities [120]. Moreover, kaempferol can inhibit PrV replication in a dose-dependent manner in vitro and exhibits higher anti-PrV activity than acyclovir, an approved therapy drug for HSV-1/2. Acyclovir displays dramatic anti-HSV activity by targeting thymidine kinase and competing with deoxyguanosine triphosphate for the viral DNA polymerase [124]. PrV inhibition by kaempferol reportedly occurs at the early stage of virus replication. In Chinese Kun Ming (KM) mice, kaempferol showed anti-PrV activity through an improved survival rate (22.22%) at 6 days-post-infection, which was higher than that of when acyclovir was administered (16.67%). Kaempferol could inhibit virus replication in several organs, especially in the brain, where viral gene copies were reduced by over 700-fold. At the same time, the pathogenic changes induced by PrV infection in the brain, lung, kidney, heart, and spleen were also alleviated [125]. Viral gene transcription is a critical step in viral replication. Immediate-early gene-, early gene-, and late gene- are the stages of PrV gene expression. PrV-only immediate-early gene IE180 is a transcriptional activator of early gene transcription [126, 127]. Research confirmed that kaempferol considerably inhibited IE180 transcription, leading to decreased transcriptional levels of the early genes EP0 and thymidine kinase. In addition, the latency-associated transcript expression was markedly reduced by kaempferol but not by acyclovir, suggesting that kaempferol could inhibit PrV latency [125].

Quercetin, another flavonoid found in vegetables, fruits, and grains and used in TCM [128], was effective against the PrV strains HNX, Ea, Bartha, and Fa in vitro. In a mouse model, quercetin plantar injection reduced mortality and PrV replication in the brain tissue, ultimately protecting the mice from the lethal challenge. Furthermore, in silico assays suggested that quercetin interacted with the PrV gD-protein and occupied most of the gD subject binding regions, inhibiting PrV adsorption by blocking nectin-1 binding to gD [129]. Intracellular reactive oxygen species (ROS) levels are generally low but can increase rapidly after virus infection, leading to oxidative stress and cell damage [130]. Quercetin could substantially reduce ROS secretion and alleviate the oxidative stress caused by PrV infection in 3D4/2 cells. According to the long non-coding RNA-micro RNA-mRNA network, this process may be related to thioredoxin-interacting protein and nitric oxide synthase 2 decrease [131].

(-)-epigallocatechin-3-gallate (EGCG), a bioactive polyphenol found in tea, has antiviral, antibacterial, antioxidant, anti-inflammatory, and antitumor activities. Recently, 50 mM EGCG could reportedly completely block viral infection at different infection multiplicities in vitro. Additionally, 40 mg/kg EGCG administered pre- and post-challenge in BALB/c mice could provide 100% protection against PrV XJ5 [132]. Additionally, dihydromyricetin extracted from *Ampelopsis grossedentata* [133], isobavachalcone, a natural chalcone compound extracted from *Psoralea corylifolia* [134], and an ethyl acetate fraction extracted from *Polygonum hydropiper* in which rutin was the most abundant compound [135], also showed antiviral activity.

### Polysaccharides

Polysaccharides are essential components and participate in maintaining biological functions. Plant polysaccharides have a wide range of sources, pharmacological effects, and a high safety, all contributing to their broad application in disease prevention and treatment. *Platycodon grandiflorus* has both edible and medicinal value, and the polysaccharides extracted from this herbal medicine (PGPSt) have an anti-PrV effect [136]. Viruses have evolved to exploit or avoid autophagy to survive by co-existing with their hosts for millions of years. Although PrV lacks the ICP34.5 gene, which can inhibit autophagy in HSV-1, recent studies revealed that autophagy inhibition is not conducive to PrV replication in mouse Neuro-2a cells in vitro [137, 138]. However, PGPSt can reduce PrV replication by inhibiting autophagosomal accumulation induced by PrV infection in vitro. Additionally, 200 µg/mL PGPSt decreased the viral gC mRNA level to 50%, and the viral proteins in PGPSt-treated PK-15 cells were considerably reduced in 12 h [136].

*Hippophae rhamnoides* polysaccharide (HRP) can enhance immunity and has antioxidant and antitumor activity [139, 140]. HRP has been suggested as a potential anti-PrV drug, which inhibits PrV adsorption and entry and reduces PrV infection-induced oxidative stress. A total of 2000 µg/mL HRP had no apparent toxicity to PK-15 cells, while 1000 µg/mL HRP almost completely inhibited viral protein gB expression. Moreover, its antiviral activity was similar to that of 10 mM lithium chloride (LiCl), a chemical drug with anti-PrV activity. However, HRP is safer than LiCl [141].

*Panax notoginseng* has various pharmacological effects, including immune modulation, neuroprotection, and antitumor activity [142]. The anti-PrV effect of *P. notoginseng* polysaccharides is mainly associated with virus adsorption and replication interferences, especially adsorption. The expression of PrV gB protein decreased substantially (98.7% inhibition rate) using 600 µg/mL P*. notoginseng* polysaccharides, but it cannot inhibit the PrV entry into PK-15 cells. Therefore, the anti-PRV mechanism of *P. notoginseng* polysaccharides needs further research [143].

*Glycyrrhiza* polysaccharide (GCP), a common Chinese medicine licorice extract, is widely used worldwide. Reportedly, 600 µg/mL GCP could completely block PrV infection. GCP’s primary antiviral effect was to inhibit PrV attachment and internalization rather than replication and release, indicating that GCP plays an inhibitory role in the early stages of PrV infection [144]. *Plantago asiatica* polysaccharide (PLP), extracted from the whole *P. asiatica* plant exerts an antiviral effect by reducing viral adsorption, entry, and intracellular ROS levels rather than affecting PrV replication and inactivation [145]. The intracellular ROS levels in PrV-infected PK-15 cells were markedly decreased and practically returned to normal after PLP treatment [145]. In addition, *Radix isatidis* polysaccharide and huaier (*Trametes robiniophila*) polysaccharide have a direct inactivation effect on PrV [146, 147]. In summary, different polysaccharides play antiviral roles in different phases of the PrV life-cycle. Thus, polysaccharide-combination therapy to increase the efficacy or reduce the toxic side effects of drugs may become a new route for PrV treatment.

In addition to flavonoids and polysaccharides, resveratrol (RES), a non-flavonoid polyphenol compound in plants and fruit, shows antiviral activity on PrV. RES has been found to inhibit PrV replication in PK-15 cells [148]. In vivo, RES protects against PrV-induced reproductive failure by recovering serum progesterone levels in mice [149]. Moreover, RES can decrease the mortality of PrV-infected piglets, inhibit viral reproduction, and improve cytokine levels [150]. The RES antiviral mechanism against PrV includes inhibiting IE180 transcriptional activation activity by targeting the Thr601, Ser603, and Pro606 sites of IE180 protein [151].

TCM extracts can also be used as vaccine adjuvants to enhance animals’ immune responses. The PrV live vaccine is mostly commercialized as a lyophilized powder diluted in saline before injection. Ginseng saponins isolated from the stem and leaves of *P. ginseng* can improve the mice’s immune response to the attenuated PrV (aPrV) vaccine as a diluent component. The aPrV vaccine containing ginseng saponins and sodium selenite (GSe) induced a considerably higher immune response in mice, and the animals showed much higher resistance to the virulent PrV challenge [152]. The function of GSe as an adjuvant was also confirmed in neonatal mice carrying the maternal antibodies (MatAbs). Despite the presence of MatAbs, the specific gB antibody and cytokine responses in neonatal mice immunized with aPrV and saline-GSe were markedly increased, and the protection of vaccinated neonates was enhanced [153]. However, researching natural drugs against PrV is mostly in vitro and the experimental animal stage. Because only a few confirmed cases have been reported, the data on natural drugs against PRV-induced human encephalitis is rare.

## Conclusions and perspectives

PR causes severe financial losses to the pig industry and considerably affects other animals with an economic interest, such as mink [154]. Furthermore, the presence of PrV in wildlife is an extensive challenge for countries that have eradicated PrV or in areas working towards eradication. In addition, PrV infection in humans requires more attention. On the one hand, as emphasized by Heldwein, “Determining how the triggering signal reaches the fusogen gB represents the next frontier in structural biology of herpesvirus entry” [45]. On the other hand, fully and correctly understanding the invasion process helps develop targeted drugs, antibodies, or blocking agents and for using genetic engineering technology to establish PrV-resistant pigs. However, several details of the PrV entry pattern remain unclear, such as the gH/L action site on gD, the entire PrV gH/L structure, the existence of a gB binding site on gD, and the existence of receptors for gH/L and gB that can mediate PrV invasion. Drugs or antibodies targeting the PrV fusion process may improve therapeutic strategies, but relevant research is scarce. The PrV-gD specific mAb 10B6 binding to gD pro-fusion domain ^316^QPAEPFP^322^, a conserved epitope, exhibited effectively neutralizing activities both in vitro and in vivo by blocking gD binding to nectin-1 [155].

Regarding gene editing, a recent study confirmed that the nectin-1/2 knockout in PK-15 cells using the CRISPR/Cas9-mediated gene targeting method conferred resistance to PrV [156]. In vivo, homozygous mutant mice with a single point amino acid mutation (F129A) in nectin-1 had milder symptoms and lower mortality than a heterozygous mutant and wild-type mice artificially infected with a highly pathogenic PrV strain. However, the homozygous mutant mice had deficient eye development [157]. According to the above results, using nectin-1 to establish animals resistant to PrV is a practical approach, although more studies are needed to determine the appropriate mutagenesis mode.

The first isolation of PrV strain hSD-1/2019 from human CSF proves that people can be infected with PRV. Due to the small number of human PrV infection reported cases, there is still a lack of clinical consensus, available treatment guidelines, and prevention experience in human PrV infection. However, a diagnostic workflow of PrV-induced human encephalitis was proposed [93]. The emergence of PrV variants is the primary reason for the classical vaccine immunization failure in pigs and contributes to diagnostic obstacles and treatment of human encephalitis. We speculate that the biological characteristics (such as virulence) of PrV variants are closely related to patients’ incubation period and disease degree. Unfortunately, there are no specific drugs for PR or PrV-induced human encephalitis. Therefore, developing antiviral drugs to prevent and treat PrV is crucial. TCM has multiple medicinal values, which also showed antiviral activity against PrV. Although research on TCM in anti-PRV primarily focuses on a single extract, TCM’s potential is undoubtedly high because TCM compounds are likely to inhibit PrV at different stages of its life-cycle. Moreover, the pathogens are less likely to develop drug resistance than single-component drugs.

In conclusion, studies of the PrV fusion mechanism and how PrV infects humans are the focus of future research. At the same time, developing natural antiviral active ingredients and effective drug targets will also be the main direction of anti-PrV drug research. Therefore, we have a long way to go to eradicate PrV.
